# Should antibiotic prophylaxis before orthopedic implant surgery depend on the duration of pre-surgical hospital stay?

**DOI:** 10.1186/s13756-018-0421-2

**Published:** 2018-11-08

**Authors:** Marie Davat, Lydia Wuarin, Dimitrios Stafylakis, Mohamed Abbas, Stephan Harbarth, Didier Hannouche, Ilker Uçkay

**Affiliations:** 1 Orthopedic Surgery Service, Geneva University Hospitals, Geneva, Switzerland; 2 Infection Control Program, Geneva University Hospitals, Geneva, Switzerland; 3 Infectiology, Balgrist University Hospital, Forchstrasse 340, 8008 Zürich, Switzerland

**Keywords:** Perioperative antibiotic prophylaxis, Implant orthopedic surgery, Length of hospital stay, Surgical site infections

## Abstract

**Background:**

Prolonged hospital stay before surgery is a risk for colonization with antibiotic-resistant microorganisms and possible antibiotic-resistant surgical site infections (SSI), which lacks acknowledgement in international guidelines for perioperative antibiotic prophylaxis.

**Method:**

Retrospective cohort study focusing on prophylaxis-resistant SSI in adult orthopedic implant patients; with emphasis on length of hospital stay prior to the index surgery.

**Results:**

We enrolled 611 cases of SSI (median age, 65 years; 241 females and 161 immune-suppressed) in four large implant groups: arthroplasties (*n* = 309), plates (*n* = 127), spondylodeses (*n* = 31), and nails (*n* = 46). The causative pathogen was resistant to the perioperative antibiotic prophylaxis regimen in 307 cases (307/611; 50%), but the length of pre-surgical hospitalization did not influence the incidences of prophylaxis-resistant SSIs. These incidences were (107/211;51%) for the admission day, (170/345;49%) within 10 days of delay, (19/35;54%) between 10 and 20 days, and (11/20; 55%) beyond 20 days of hospital stay before surgery. The corresponding incidences of methicillin-resistant staphylococci were 13%, 14%, 17%, and 5%, respectively. In adjusted group comparisons, the length of prior hospital stay was equally unrelated to future prophylaxis-resistant SSI (odds ratio 1.0, 95% confidence interval 0.99–1.01).

**Conclusions:**

In our retrospective cohort of orthopedic implant SSI, the length of pre-surgical hospital stay was unrelated to the incidence of prophylaxis-resistant pathogens.

## Background

Prolonged hospital stay before orthopedic surgery is a potential risk for acquisition of antibiotic-resistant microorganisms [1]. Thus in case of delayed surgery and surgical site infections (SSI) [2], the SSI might be multi-resistant or resistant to the perioperative antibiotic prophylaxis that was administered during the index operation. For example in our hospital, lengthening of hospital stay by one additional day was associated with a 5% increment of new MRSA carriage [1]. Moreover, prophylaxis-resistant germs may remain undetected except for outbreak situations or scientific studies [3]. This possible threat not only concerns methicillin-resistant *Staphylococcus aureus* (MRSA) [2, 3], but also methicillin-resistant coagulase-negative staphylococci [4], cephalosporin-resistant enterococci [5], non-fermenting Gram-negative rods [6] or extended-spectrum β-lactamase (ESBL) producing rods [7]. These pathogen groups all escape to standard prophylaxis with first-and second generation cephalosporins [2]. Especially, implant-related orthopedic surgery [8] is prone to SSI by methicillin-resistant staphylococci [4].

Many colleagues administer vancomycin or other broad-spectrum agents as prophylaxis, alone or in combination, for patients with long hospital stays (*personal communication*). There are no data supporting this practice. National [9] and international [10] guidelines and consensus meetings [11] do not provide robust evidence on the choice of the prophylactic agent upon the length of pre-surgical stay. Indeed, international experts unanimously advocate single-dose cephalosporins or vancomycin for any orthopedic procedures [9–12].

In this retrospective cohort analysis, we specifically link the duration of pre-surgical hospital stay to the antibiotic resistance profile of orthopedic implant-related SSIs. We do not compute SSI risks or report treatment successes that we already have published elsewhere [13–15].

## Methods

The Geneva University Hospitals is a tertiary center with a long tradition of clinical research regarding prevention of orthopedic implant-related infections [8]. The most recent prevalence of methicillin-resistant *Staphylococcus aureus* (MRSA) and methicillin-resistant coagulase-negative staphylococci among the clinical isolates in the orthopedic service were1% [13] and 75% [4, 16], respectively. The hospital recommends a single intravenous dose of pre-operative cefuroxime 1.5 g as standard prophylaxis in orthopedic surgery and traumatology. Only for cases with convincing history of penicillin allergy [17] or past/present MRSA carriage [3], we recommend one dose of vancomycin 1 g intravenously. Discipline regarding these recommendations is very good, with only maximal 5–10% deviations according to the last control assessments. We currently lack a univ ersal policy for searching and decolonizing *S. aureus* body carriage before surgery. Positive urinary or anal carriage of ESBL [7] does not alter our recommendations for orthopedic surgery. If surgery lasts for more than 4 h, prophylaxis is repeated. In selected cases, surgeons may continue it up to 24 h; except for open fractures with longer durations of preemptive treatment [18]. Since 2016, the standard dose was doubled for obese patients with more than 100 kg weight [19]. In selected cases, surgeons also implement arthroplasties with or without aminoglycoside-containing cement.

For the actual study, we used a composite database (Ethical Committee no. 13–178, 08–057 [13], 08–061 [20], and 14–198**)**, including all adult patients with orthopedic implant SSI [8] and a minimal follow-up of two years [1]. We excluded cases that were amputated [21], orthopedic surgery cases without implants, community-acquired infections, recurrent episodes of the same infection and all patients necessitating actual or recent systemic antibiotic administration during the last 2 weeks. Our SSI definitions were based on the Center of Disease Control standards [22]. Basically, any infection within 1 year of implantation was a SSI, unless proven otherwise; e.g. by clear evidence of a hematogenous [23] or lymphogenous origins [24]. We defined hospital stay as a hospitalization in acute care settings. Consequently, we considered long-term care facilities not as hospitals. We collected several microbiological samples from pus or deep intraoperative tissues, and ignored results of superficial specimens or of a sinus tract. The microbiology laboratory processed all specimens according to Clinical and Laboratory Standard’s Institute recommendations [25], before switching to the EUCAST criteria (European Committee) in 2014 [26].

### Statistical analyses

Our primary objective was to assess the association between the length of prior hospital stay to future SSIs that were resistant to standard antibiotic prophylaxis (cefuroxime or vancomycin). We performed group comparisons using the Pearson-χ ^2^ or the Wilcoxon-ranksum-test. An unmatched multivariate logistic regression analysis determined associations with the outcome “SSI resistant to standard prophylaxis”. We introduced independent variables with a *p* value ≤0.05 in the univariate analysis stepwise into the multivariate analysis, except for length of prior hospital stay, which we forced into the final model. We arbitrarily categorized the length of hospital stay individually for the days 0, 1, 2, 3, 4, for the groups between 5 and 9 days, 10–20 days, and more than 20 days; and plotted them against the occurrence of resistant SSIs. We used STATA software (9.0, STATA™, USA) and considered *p* values ≤0.05 (two-tailed) as significant.

## Results

We included 611 orthopedic SSI cases meeting our study criteria (among 611 patients, 241 females (39%) and including 161 immune-suppressed persons (27%): diabetes mellitus (*n* = 73), active cancer (40), severe chronic alcoholism (25), medicamentous immune-suppression (20), cirrhosis CHILD C (11), dialysis (4), solid organ transplantation (1), or a combination of different immune-suppressed states. Upon diagnosis, the median age of the patients was 65 years and the median serum C-reactive protein levels were 83 mg/L. The presence of soft-tissue abscesses and bacteremia complicated the infections in 73 (12%) and 98 (16%) episodes, respectively. The infected implants were: arthroplasties (*n* = 309), plates (127), spondylodeses (31) [27], nails (46) [15], and various others (98). In 25 episodes, the infection occurred in the foot. Our laboratory detected 84 different microbiological constellations with the five most frequently identified pathogens being *Staphylococcus aureus* (*n* = 166), streptococci (46), Gram-negatives [6] (140; with 80 non-fermenters including 42 *Pseudomonas aeruginosa* cases, and 24 anaerobes [28]), enterococci [5] (33), *S. lugdunenssis* [16] (9), and skin commensals (134)*.* In 100 cases, SSI were polymicrobial and in 35 cases culture-negative [29].

### Associations with previous hospital stay

Overall, 556 (90%) implant surgeries with subsequent SSI were performed within 10 days after admission, 35 between 10 to 20 days, and 20 after 20 days since admission. Formally, the study population was already hospitalized during a median delay of 1 day (range, 0–178 d, interquartile range, 0–3 d) before the index surgery, and 44 patients had previously known MRSA carriage [3]. The prophylactic regimens followed the institutional standards for the majority of cases, but we detected the following deviations: several doses of cefuroxime (*n* = 30), use of ciprofloxacin (1), amoxicillin/clavulanic acid (1), clindamycin (1), and cefazolin (1). Overall, 36 patients received vancomycin, of which 28 episodes because of previous positive MRSA carriage in the past, and 8 probably because of presumed betalactam allergy or betalactam intolerance of various nature. In contrast, 16 former MRSA carriers lacked vancomycin for which the reasons were unknown. In 33 episodes (5%), we ignored the agent of prophylaxis, which was classified as “usual” according to the records.

**Fig. 1:**
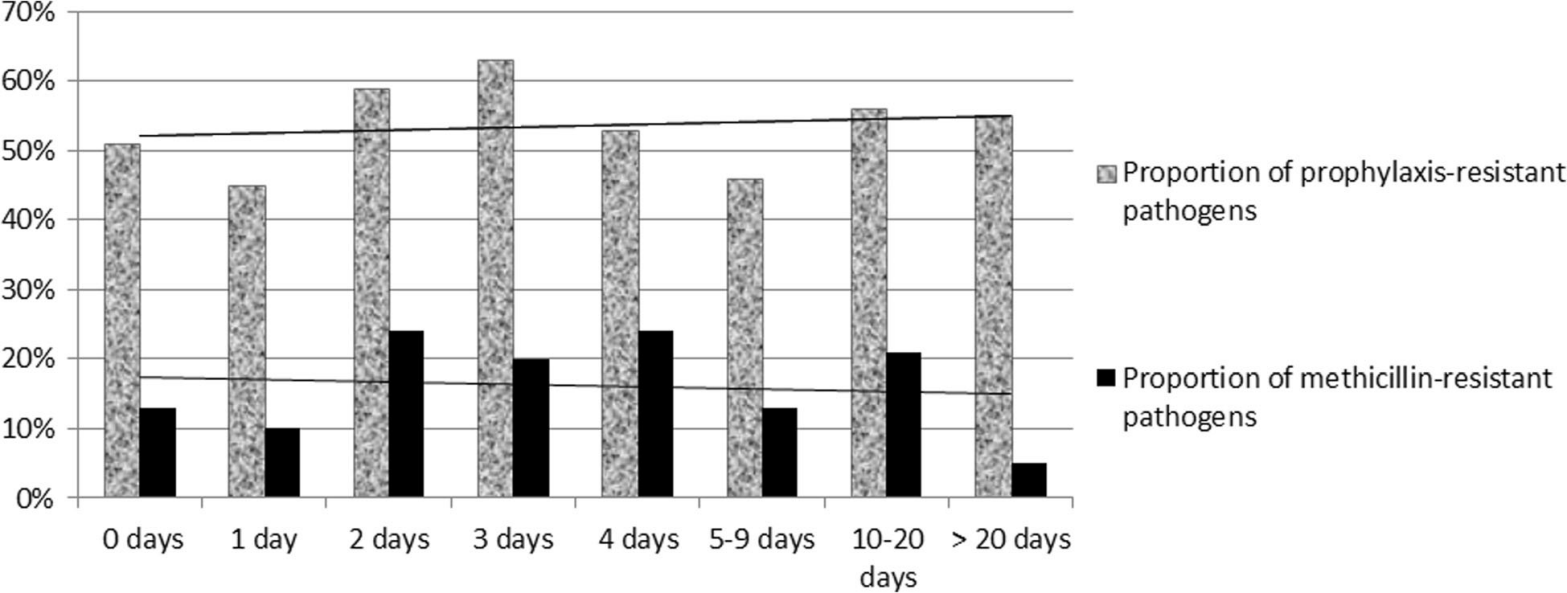
Proportions of future resistant pathogens to standard prophylaxis (vertical axis) according to the delay between admission and the index implant surgery (horizontal axis). The corresponding trend lines are almost horizontal

Overall, the causative pathogen of future SSI was resistant to prior prophylaxis in 307 cases (307/611; 50%) (Table 1), but lacked association to previous length of pre-surgical hospitalization. The incidences of resistant pathogens were (107/211; 51%) for surgeries performed on the day of admission, (89/200; 45%) on Day 1, (24/41; 59%) on Day 2, (25/40; 63%) on Day 3, (9/17; 53%) on Day 4, (18/39; 46%) between 5 and 9 days since admission, (24/43; 56%) between 10 and 20 days, and (11/20; 55%) beyond 20 days after admission. The corresponding incidences of SSI due to methicillin-resistant staphylococci were 13% (27/211), 10% (19/200), 24% (10/41), 20% (8/40), 24% (4/17), 13% (5/39), 21% (9/43) and 5% (1/20), respectively. Figure 1 display the proportion graphically and denies the existent of a threshold in the number of days of hospital stay prior to surgery and subsequent prophylaxis-resistant and methicillin-resistant SSIs. The proportion of aminoglycoside-resistant SSI (that we computed because of possible cemented arthroplasty) was very low (3 of 611 cases; 1%). A (past) history of MRSA and lack of vancomycin prophylaxis was the only variable related to multi-resistant SSI.

**Table 1:** Comparisons of demographic and clinical variables of adult orthopaedic implant patients with future surgical site infections resistant to the prophylactic regimen of the index surgery versus prophylaxis-susceptible surgical site implant infections

	Susceptible to prior prophylaxis		Resistant to prior prophylaxis
Total *n* = 611	*n* = 304	*p* value*	*n* = 307
Female sex	118 (39%)	0.752	123 (40%)
Age (median)	67 years	0.097	62 years
Past history of MRSA^b^ body carriage	12 (4%)	***0.002***	32 (10%)
Immune suppression^a^	91 (30%)	***0.030***	70 (23%)
- diabetes mellitus	45 (15%)	***0.045***	28 (9%)
Shoulder implants	12 (4%)	0.235	7 (2%)
Arthroplasties	147 (48%)	0.275	162 (53%)
Spondylodeses	14 (5%)	0.600	17 (6%)
Plates	66 (22%)	0.575	61 (20%)
Intramedullary nails	20 (7%)	0.376	26 (8%)
Foot osteosyntheses	15 (5%)	0.295	10 (3%)
Duration of prior hospital stay (median)	1 day (range, 0–178 d)	0.408	1 day (range, 0–68 d)

*Significant *p* values ≤0.05 are displayed ***in bold and italic***

^a^Immune suppression = diabetes mellitus, corticosteroid medication, organ transplantation, cirrhosis CHILD C, dialysis, or active cancer

^b^Methicillin-resistant *Staphylococcus aureus*

### Multivariate adjustment

In view of the considerable case-mix, we performed an adjusted logistic regression analysis. Here, the length of prior hospital stay was equally unrelated to prophylaxis-resistant SSI as a continuous variable (odds ratio 1.0, 95% confidence interval 0.99–1.01) or when breaking down to various stratifications (Table 2). Also, in this regression analysis, and unlike the former crude group comparison, immune-suppression lacked associations with prophylaxis-resistant SSI. The goodness-of-fit testing of our final model was non-significant (*p* = 0.31).

**Table 2:** Univariate and multivariate analyses of factors potentially related to antibiotic prophylaxis-resistant surgical site infections *(Logistic regression analysis; results expressed as odds ratios with 95% confidence intervals)*

Total *n* = 611	Univariate analysis	Multivariate analysis
Female sex	1.0, 0.7–1.4	n.d.
Age	1.0, 1.0–1.0	1.0, 1.0–1.0
Past history of MRSA^b^ body carriage	***1.3, 1.1–2.9***	***1.4, 1.1–3.3***
Immune suppression^a^	0.7, 0.5–1.1	n.d.
- Diabetes mellitus	0.7, 0.4–1.2	n.d.
Shoulder implants	0.5, 0.2–1.5	n.d.
Arthroplasties	1.1, 0.8–1.8	1.2, 0.8–1.9
Spondylodeses	1.4, 0.7–2.9	1.4, 0.6–3.1
Plates	1.0, 0.6–1.5	n.d.
Intramedullary nails	1.2, 0.6–2.3	1.3, 0.6–2.6
Foot osteosyntheses	0.7, 0.3–1.6	0.8, 0.3–1.9
Duration of prior hospital stay	1.0, 1.0–1.0	1.0, 1.0–1.0
- 10–20 days compared to ≤10 days	1.1, 0.5–2.4	1.2, 0.5–2.5
- ≥ 20 days compared to ≤10 days	1.0, 0.6–1.7	1.1, 0.4–3.3

*n d.* = not done

^*^Statistically significant results are displayed ***in bold and italic***

^a^Immune suppression = diabetes mellitus, corticosteroid medication, organ transplantation, cirrhosis CHILD C, dialysis, or active cancer

^b^Methicillin-resistant *Staphylococcus aureus*

## Discussion

In our cohort of orthopedic implant infections among adult patients, the length of hospital stay before the index surgery (implantation) was unrelated to the risk of future SSI due to prophylaxis-resistant pathogens such as methicillin-resistant staphylococci [4, 13]. Therefore, we argue against the broadening of the antibiotic prophylaxis with second-generation cephalosporins towards combinations that include more Gram-positive or Gram-negative coverage. One might argue, whether the study question is of importance. International recommendations are clear and do not recommend this [1, 2]. And yet, according to our experience, many surgeons or physicians often broaden the prophylaxis against official recommendations. Regarding prophylactic issues, our center shows high compliance and the study population is homogenous. Hence, we could easily perform tour study by avoiding major confounding and substantial interactions, making the interpretations more difficult.

In the literature, many research groups investigated the influence of a delay between admission and surgery with the occurrence of subsequent infection and its pathogen profile. However, these studies concerned open fractures, with time delays ranging from 0 to 24 h [18]. We found only one study specifically linking longer hospitalization delays with healthcare-associated infections [30]. In this study from Brazil, patients with nosocomial infections due to MSSA (not only orthopedic implants) revealed a median delay of prior hospital stay of 9 days, compared to MRSA infections with a past median delay of 18 days [30].

In contrast, the literature is full of opinion papers and retrospective studies investigating the possibility of better outcomes with broader prophylaxis. The propositions of the authors differ from one paper to another and focus on different strategies which are: continuing the prophylaxis beyond a single dose [31, 32], augmenting of doses [19], combining with local prophylaxis [33, 34] (especially local vancomycin in spine surgery [35]), double prophylaxis [36] against Gram-negative [37], Gram-positive [38, 39], methicillin-resistant strains [4] and anaerobes [28], or by investigating the performance of universal glycopeptid prophylaxis [40–42]. In summary, the majority of these enhancements failed to reduce SSI risk, at least not in orthopedic surgery [34, 39–42]. Exceptions remain rare [32, 37], very specific and often not reproducible by other research groups [12, 31, 36]. Branch-Elliman et al. estimated that the number of orthopedic cases needed to prevent one Gram-positive SSI with vancomycin would be 1:53 for known MRSA carriers, compared to 1:176 for unknown carriers [39]. Also, concomitant colonization with MRSA does not always protect from colonization by susceptible *S. aureus* (MSSA) strains [1].

Broader antibiotic prophylaxis can be harmful, especially with prophylactic  aminoglycosides against Gram-negative pathogens [38, 39]. Numerous studies reported transient kidney injuries by aminoglycosides [39] or combined vancomycin prophylaxis [12] in orthopedic surgery. The risk for antibiotic-resistant organisms seems to be negligible [35]. Walker et al. reported that following a change in prophylaxis (from floxacillin & gentamycin to amoxicillin/clavulanic acid), they witnessed a 63% decrease in postoperative renal insufficiencies [43]. In 2009, Scotland issued a target to reduce *Clostridium difficile* outbreaks. Consequently, hospitals changed prophylaxis from a cephalosporin to gentamicin-containing regimens (4 mg/kg), resulting in a 94% increase in kidney injuries [38].

Besides the fact that our study is retrospective, it has several limitations. First, we excluded already infected cases. Hence, we can only address the risk of prophylaxis-resistant SSI, but we cannot compare between infected and non-infected populations, or with patients who were under systemic antibiotic selection for any reason. Second, our orthopedic service has no policy of pre-surgical *S. aureus* [2] decolonization. Such a strategy might alter the association between the length of hospital stay and the patterns of subsequent SSI. Third, we assume like many other colleagues that most SSIs origin in the operating theatre [2], and that the length of hospital stay is a surrogate of nosocomial acquisitions of resistant pathogens. In our daily clinical practice, we usually neglect to assess individual colonization throughout the hospital stay. This assumption might not be granted. Studies from Sweden suggest a mounting colonization pressure by methicillin-resistant staphylococci after two to three days [4] post-admission, and ESBL acquisitions in orthopedic wards has also been demonstrated [7], i.e. during wound care or wound breakdowns. Regarding these ward-born SSIs, the perioperative antibiotic prophylaxis during the index surgery would naturally lack influence; which we equally cannot control for in our retrospective analysis. Fourth, in our tertiary center, we use cefuroxime as standard antibiotic prophylaxis for many surgical disciplines, instead of cefazolin, which is another recommended agent in most guidelines for orthopedic surgeries. Most experts would agree that the difference and ecological impact between the two second-generation cephalosporins would be minimal, as they are close molecules in terms of clinical spectrum and efficacy. Although we cannot formally pronounce on the hypothetical results of our study performed under cefazolin, we nevertheless would expect the same results. Fifth, most of our patients had surgery within few days since admission, with a median delay of only 1 day. From a microbiological point of view, it seems rather unlikely that such a short hospitalization changes colonization with antibiotic-resistant germs. Nevertheless, we intended to study real-life conditions without selection biases. Theoretically, we could have performed a study only with those patients operated after 1 week of hospital stay or longer. This would, however, introduce a major bias and a very small and too specific study population, which we avoided. Sixth, many our implant infections were due to coagulase-negative staphylococci and other skin commensals. Usually, these bacteria are often regarded as contaminants. In our study, all bacterial results stem from several deep intraoperative tissue specimens, making contamination unlikely. Moreover, skin commensals, including coagulase-negative staphylococci and especially *S. epidermidis*, are frequent pathogens of low-grade orthopedic implants [8, 14–16] due to their ability to perform biofilms [44]. Lastly, we cannot retrospectively enumerate the individual reasons for a delayed surgery. As simple as it seems, in a retrospective study this question is very difficult to be answered. The reasons may vary between lack of operation slots, triage issues, lack of patient’s and family’s consent, nosocomial fractures occurring during a hospital stay for another reason, availability of the individual surgeon, week-ends and holidays, non-availability of the specific osteosynthesis material, or a panoply of combined reasons. However, we do not think that the individual reason for delay would have influenced our findings in this large epidemiological study.

## Conclusions

According to our retrospective cohort analysis, a long pre-surgical hospital stay was not associated with more prophylaxis-resistant SSIs, in 611 adult patients undergoing orthopedic implant surgery, when compared to those with prophylaxis-susceptible pathogens. We keep our antibiotic perioperative prophylaxis policy as it is, regardless of the duration of pre-surgical length of hospital stay.
